# LRG1, a novel serum biomarker for iMCD disease activity

**DOI:** 10.1186/s40364-025-00767-1

**Published:** 2025-04-07

**Authors:** Miao-yan Zhang, Zi-han Yang, Yu-chong Qiu, Yu-han Gao, Si-yuan Li, Yue Dang, Lu Zhang, Jian Li

**Affiliations:** Department of Hematology, Peking Union Medical College Hospital, Chinese Academy of Medical Sciences and Peking Union Medical College, No. 1 ShuaifuyuanNo. 1 Shuaifuyuan, Dongcheng District, Beijing, 100730 China

**Keywords:** Leucine-rich alpha-2-glycoprotein-1 (LRG1), Idiopathic Multicentric Castleman Disease (iMCD), Biomarker, Proteomics

## Abstract

Idiopathic multicentric Castleman disease (iMCD) is a rare lymphoproliferative disorder characterized by systematic inflammatory symptoms and multiorgan dysfunction caused by a cytokine storm. The current assessment of treatment response in iMCD lack sensitivity due to the heterogeneity of clinical features. We performed proteomic analysis using Data Independent Acquisition (DIA) mass spectrometry (MS) on 33 serum samples in different disease states from 17 patients. Leucine-rich alpha-2-glycoprotein-1 (LRG1) emerged as one of the proteins with most significantly different expression, exhibiting lower levels in response to treatment. Enzyme-linked immunosorbent assay (ELISA) on a larger cohort of 146 serum samples (96 disease flare, 28 biochemical partial response, 22 biochemical complete response) from 100 iMCD patients further confirmed this association, demonstrating a significant decrease in serum LRG1 level following successful treatment. Notably, LRG1 remained elevated in patients with ongoing inflammation during siltuximab therapy when CRP failed to accurately reflect disease activity. Additionally, serum CRP/LRG1 ratio differed across iMCD subtypes, suggesting potential variations in inflammatory pathways. These findings support serum LRG1 as a valuable biomarker for iMCD disease treatment response and activity, and may provide insights into underlying disease mechanisms.

**Supplementary Information:**

The online version contains supplementary material available at 10.1186/s40364-025-00767-1.

To the editor

Idiopathic multicentric Castleman disease (iMCD) is a rare lymphoproliferative disorder characterized by systemic inflammation and multiorgan dysfunction caused by a cytokine storm [1]. It exhibits significant clinical heterogeneity, with subtypes including iMCD-TAFRO [2] and iMCD-idiopathic plasmacytic lymphadenopathy (IPL) [3, 4] showing distinct symptoms and prognoses. Current assessment of treatment response relies on symptoms, biochemical markers and lymph node size [5], but these lack sensitivity due to the disease's heterogeneity. Thus, identifying sensitive biomarkers is crucial for enhancing the evaluation of treatment efficacy.

While therapies targeting IL-6, a key driver of the cytokine storm, have shown promise, [6] the lack of universal response suggests the involvement of additional inflammatory pathways. C-reactive protein (CRP), primarily stimulated by IL-6, [7] may not reliably indicate disease activity in non-responders, highlighting the need for alternative biomarkers, especially during anti-IL-6 therapies. Methods and materials of this study can be found in the supplementary material.

To identify biomarkers associated with disease activity, we performed LC–MS/MS and DIA proteomic analysis on 33 serum samples from 17 iMCD patients (16 Flare, 4 PR, and 13 CR; Supplementary Table 1). We detected and quantified 517 proteins, with 130 differentially expressed between Flare and CR (Fig. 1A; Supplementary Table 2). Leucine-rich alpha-2-glycoprotein-1 (LRG1) exhibited a consistent change across Flare, PR, and CR stages, with the most significantly different expression (Supplementary Table 3).

**Fig. 1:**
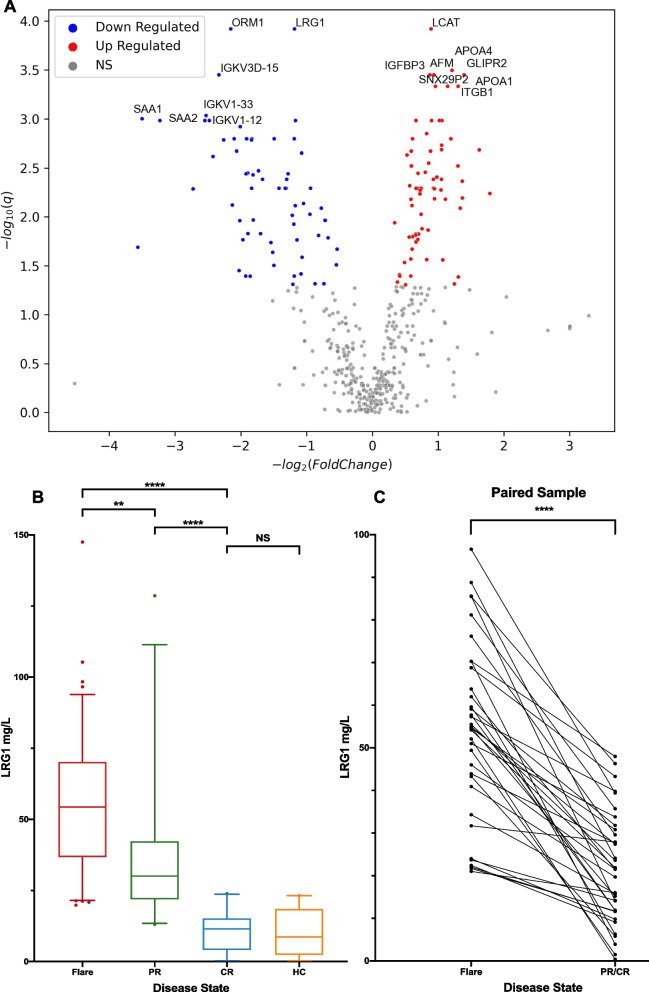
**A** Volcano plot on comparing serum protein levels between Flare and CR samples. 64 proteins were significantly up-regulated in CR compared to Flare (red dots), and 66 proteins significantly down-regulated (blue dots). Leucine-rich alpha-2-glycoprotein-1 (LRG1) was one of the proteins with most significantly different expression. **B** The average serum LRG1 concentration was highest in Flare samples (54.5 ± 24.2 mg/L), followed by PR samples (36.2 ± 23.6 mg/L), and lowest in CR samples (10.6 ± 7.2 mg/L). Statistical analysis revealed significant differences between all three groups (Flare vs PR, *p* = 0.0023; Flare vs CR, *p* < 0.0001; PR vs CR, *p* < 0.0001). Serum LRG1 levels in iMCD patients who achieved CR were not significantly different from those in healthy controls (10.6 ± 7.2 mg/L vs 9.8 ± 8.4 mg/L, *p* = 0.9997). **C** A paired-sample analysis of 35 patients showed a significant decrease in serum LRG1 level following treatment. Patients in Flare exhibited a mean reduction of 32.3 ± 18.6 mg/L (*p* < 0.0001) after achieving PR or CR. The data are presented as mean ± SD. **P < 0.01, ****P < 0.0001, by Brown-Forsythe and Welch ANOVA and Dunnett's T3 test (B.) and paired t test (C.). NS, not significant

Then we performed ELISA to quantify LRG1 levels in 146 samples from 100 iMCD patients (96 Flare, 28 PR, and 22 CR samples) and 22 healthy controls (HC). Serum LRG1 decreased significantly in response to treatment (Fig. 1B, C). On the other hand, serum LRG1 levels in CR samples were not significantly different from those in HC, suggesting that LRG1 levels normalize during disease remission.

We serially measured serum LRG1 in 6 iMCD patients undergoing siltuximab treatment (Fig. 2). LRG1 levels declined gradually with disease activity, aligning with other biochemical markers (ALB, HGB, and eGFR). In contrast, CRP levels dropped sharply post-siltuximab. Notably, in a non-responding patient (P6), CRP was suppressed, while LRG1 remained elevated, indicating ongoing disease activity.

**Fig. 2:**
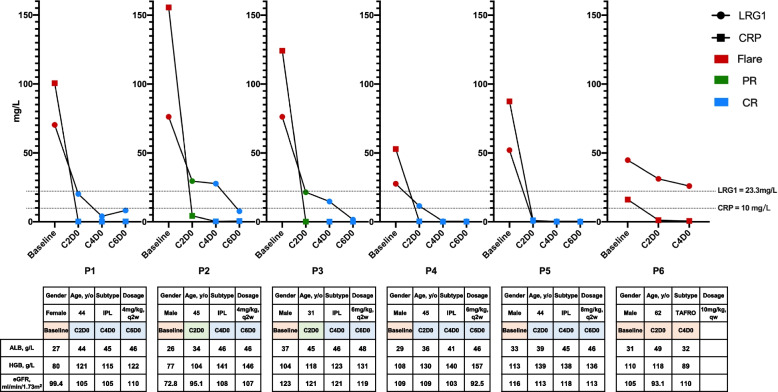
Serum LRG1 and CRP levels in 6 iMCD patients during siltuximab therapy. Serum LRG1 levels demonstrated a gradual decline corresponded with disease activity, as indicated by other biochemical markers for iMCD treatment response including ALB, HGB, and eGFR. On the other hand, CRP levels showed a sharp decline following siltuximab administration. Due to the absence of a universally accepted normal range for serum LRG1, we established a reference threshold of 23.3 mg/L (95th percentile of healthy controls). HGB, hemoglobin; ALB, albumin; eGFR, estimated glomerular filtration rate; qw, once a week; q2w, once every two weeks

To explore potential differences in inflammatory profiles across iMCD subtypes, we compared serum levels of LRG1, CRP, and IL-6, along with the CRP/LRG1 ratio, in iMCD patients during disease flare (26 iMCD-TAFRO, 37 iMCD-IPL, and 33 iMCD-NOS without IPL) (Supplementary Fig. 1). Our results revealed significantly higher levels of LRG1, CRP and IL-6 in iMCD-IPL compared to iMCD-NOS without IPL and iMCD-TAFRO during disease flare. Meanwhile, the CRP/LRG1 ratio was highest in iMCD-IPL.

LRG1, an acute-phase protein elevated in various inflammatory conditions including autoimmune diseases [8, 9], infections [10], and malignancies, [11] is associated with inflammatory burden, supporting its potential as a biomarker in iMCD, which is characterized by cytokine storm and systemic inflammation. Our study demonstrated a consistent decrease in LRG1 levels following successful treatment, suggesting its utility in monitoring iMCD treatment response. Unlike CRP, which is primarily stimulated by IL-6, LRG1 responds to a broader range of cytokines, including IL-6, OSM, TNFα, IL1-β, and TGF-β [12]. Previous studies suggest LRG1 may be a more reliable inflammatory biomarker during IL-6-targeted therapies, as CRP levels often fail to accurately reflect disease activity or infections [9]. Our findings further support this notion, showing that LRG1 levels corresponded with disease activity in siltuximab-treated iMCD patients. These results highlight the potential of LRG1 as a more sensitive indicator of disease activity complementary or alternative to CRP, particularly in the context of siltuximab therapy. However, the limited data on siltuximab-treated patients may restrict the generalizability of our findings. Validation in larger, independent cohorts and establishment of reference values for LRG1 in iMCD are necessary before incorporating LRG1 into the iMCD biomarker panel.

iMCD presents with highly heterogeneous clinical features across subtypes. The higher levels of LRG1, CRP and IL-6 in iMCD-IPL align with previous reports suggesting a more prominent systemic inflammation in this group [4]. Given that LRG1 responds to a broader range of cytokine stimulation than CRP, which is primarily IL-6-induced, the highest CRP/LRG1 ratio may suggest a more prominent role for IL-6 in iMCD-IPL pathogenesis. It also indicates the potential involvement of additional inflammatory pathways beyond IL-6 in iMCD-TAFRO and iMCD-NOS without IPL.

Taken together, serum LRG1 is a valuable biomarker for iMCD disease treatment response and activity, and may provide insights into underlying disease mechanisms.

## Supplementary Information

Supplementary Material 1.

Supplementary Material 2.

Supplementary Material 3.

Supplementary Material 4.

Supplementary Material 5.

## Data Availability

The datasets used and analyzed during the current study are available from the corresponding author on reasonable request.

## Abbreviations

iMCD: Idiopathic multicentric Castleman disease
TAFRO: Thrombocytopenia, anasarca, fever, reticulin fibrosis, renal impairment, and organomegaly
IPL: Idiopathic plasmacytic lymphadenopathy
IL-6: Interleukin-6
CRP: C-reactive protein
LC–MS/MS: Liquid chromatography coupled with tandem mass spectrometry
DIA: Data-independent acquisition
Flare: Untreated newly-diagnosed patient and treated patient whose conditions did not meet the criteria for PR or CR
PR: Biochemical partial response
CR: Biochemical complete response
HC: Healthy control
LRG1: Leucine-rich alpha-2-glycoprotein-1
ELISA: Enzyme-linked immunosorbent assay
ALB: Albumin
HGB: Hemoglobin
eGFR: Estimated glomerular filtration rate
