# Supplementary material for: LRG1, a novel serum biomarker for iMCD disease activity
Source: Biomark Res. 2025 Apr 7;13:56. doi: 10.1186/s40364-025-00767-1 (PMC11974158; doi:10.1186/s40364-025-00767-1)
Supplement: Supplementary file 1 — Supplementary Material 1. [file 40364_2025_767_MOESM1_ESM.docx]

**The Supplementary Methods**

**Patients and serum samples**

We collected clinical data and serum samples from iMCD patients in flare and remission states diagnosed between August 2015 and December 2023 at Peking Union Medical College Hospital. Laboratory measurements including hemoglobin (HGB), serum albumin (ALB), estimated glomerular filtration rate (eGFR) and serum C-reactive protein (CRP) on the same day serum samples were taken and IL-6 at baseline were retrieved from clinical record. iMCD diagnosis were made according to Castleman Disease Collaborative Network (CDCN) consensus criteria. iMCD-TAFRO, iMCD-IPL and iMCD-not otherwise specified (NOS) without IPL were defined as described by Nishimura et al. ^[2]^ and Zhang et al. ^[3]^, respectively. In our study, disease response to treatment was assessed according to biochemical response defined by CDCN consensus criteria. Biochemical complete response (CR) was defined as the normalization of laboratory tests, including HGB, ALB, eGFR, and CRP. Biochemical partial response (PR) was defined as an improvement of more than 50% in these biochemical tests. Untreated newly-diagnosed patient and treated patient whose conditions did not meet the criteria for PR or CR were classified as experiencing a disease flare (Flare).

**Mass-spectrometry-based proteomics analysis**

All serum samples for proteomic analysis were depleted with top 14 high-abundant depletion spin columns (Thermo Scientific™) according to the manufacturer’s protocol. The depleted serum samples were subjected to in-solution digestions with Lys-C and trypsin. SOLAµTM HRP plate (Thermo Scientific™) was used for desalting and ionic detergent-removal. The peptides were dissolved to 0.5 μg/μl with 0.1% formic acid, and then subjected to liquid chromatography coupled with tandem mass spectrometry (LC-MS/MS) analysis. Data-independent acquisition (DIA) proteomic analysis was performed using a Thermo LC-20 AD nano-HPLC (Thermo Fisher Scientific) with Orbitrap Fusion Lumos™ quadrupole-electrostatic field orbitrap high resolution mass spectrometry (Thermo Fisher Scientific, USA), and data acquisition and processing were managed by software XCalibur 4.3 (Thermo Fisher Scientific). The analysis was performed following the manufacturer’s instructions. The DIA data were analyzed using the Skyline software package. Results were then imported into MSstats (3.21.3). We compared protein expression profiles between Flare, PR, and CR samples. Benjamini-Hochberg method was applied to adjust the p-values and calculate the false discovery rate (FDR). Proteins with a fold change >1.5 and an adjusted p-value (q-value) < 0.05 were considered to be differentially expressed proteins (DEPs). Proteins exhibiting consistent directionality of change (i.e., upregulation or downregulation) from Flare to PR and from PR to CR were selected. Among these proteins, we further identified those that showed significant differential expression in pairwise comparisons between Flare vs. PR, PR vs. CR, and Flare vs. CR. After applying Benjamini-Hochberg method to control the FDR, no proteins met the significance threshold across all pairwise comparisons. However, prior to correction, LRG1 was the only protein that showed significant differential expression across all disease stages.

**Quantification of LRG1 by enzyme-linked immunosorbent assay (ELISA)**

ELISA was used to quantify LRG1 levels in 146 serum samples from 100 iMCD patients in different disease states, and 23 samples serially obtained from 6 iMCD patients during siltuximab therapy to further evaluate LRG1's potential as a biomarker for iMCD disease activity and response to treatment. Sera from 22 healthy volunteers without any diagnosed disease were used as samples of healthy controls. The levels of LRG1 were measured using the Human LRG1 ELISA Kit (Abcam, ab260066) following the manufacturer’s instructions. Absorbance measurements were read using an iMARK microplate reader (BIO-RAD).

**Statistical Analysis**

Statistical analysis was performed using GraphPad Prism (version 8.3.1, GraphPad Software, La Jolla, CA, USA). We used independent or paired samples t-test or Welch's corrected t-test when comparing two groups. We used ordinary one-way ANOVA and Tuckey’s test, Brown-Forsythe and Welch ANOVA and Dunnett's T3 test, or Kruskal-Wallis’s test and Dunn’s test for multiple comparisons.

**Legends for the supplementary tables and figures**

**Supplementary Table 1.** Characteristics of iMCD patients in the proteomic cohort.

**Supplementary Table 2.** List of differentially expressed proteins comparing CR to Flare.

**Supplementary Table 3.** All identified proteins and their statistical significance in differential expression analysis.

**Supplementary Figure 1.** Inflammatory feature differs among iMCD subtypes. (A.) Serum LRG1 level was significantly higher in iMCD-IPL compared to iMCD-NOS without IPL and iMCD-TAFRO (64.5 ± 26.0 mg/L vs. 49.8 ± 18.8 mg/L, p = 0.025, and vs. 46.3 ± 23.6 mg/L, p = 0.008). The difference between iMCD-NOS without IPL and iMCD-TAFRO was not significant. (B.) CRP was highest in iMCD-IPL (130.5 ± 56.8 mg/L), followed by iMCD-NOS without IPL (59.2 ± 41.5 mg/L), and lowest in iMCD-TAFRO (18.5 ± 14.5 mg/L) (p < 0.0001). (C.) IL-6 was highest in iMCD-IPL (41.6[21.3-63.6] pg/ml), followed by iMCD-NOS without IPL (15.7[7.6-43.4] pg/ml) and lowest in iMCD-TAFRO (7.7[5.2-11.4] pg/ml) (p < 0.0001). (D.) CRP/LRG1 was highest in iMCD-IPL (2.11 ± 0.77), followed by iMCD-NOS without IPL (1.05 ± 0.51) and IMCD-TAFRO (0.38 ± 0.29) (p < 0.0001).

Data expressed as mean ± standard deviation or median (range or interquartile range [IQR]).

*P <0.05, **P <0.01, ****P < 0.0001, by ordinary one-way ANOVA and Tuckey’s test (A.), Brown-Forsythe and Welch ANOVA and Dunnett's T3 test (B. and D.), and Kruskal-Wallis’s test and Dunn’s test (C.). NS, not signiﬁcant.
