# Supplementary figures and images for: LRG1, a novel serum biomarker for iMCD disease activity
Source: Biomark Res. 2025 Apr 7;13:56. doi: 10.1186/s40364-025-00767-1 (PMC11974158; doi:10.1186/s40364-025-00767-1)

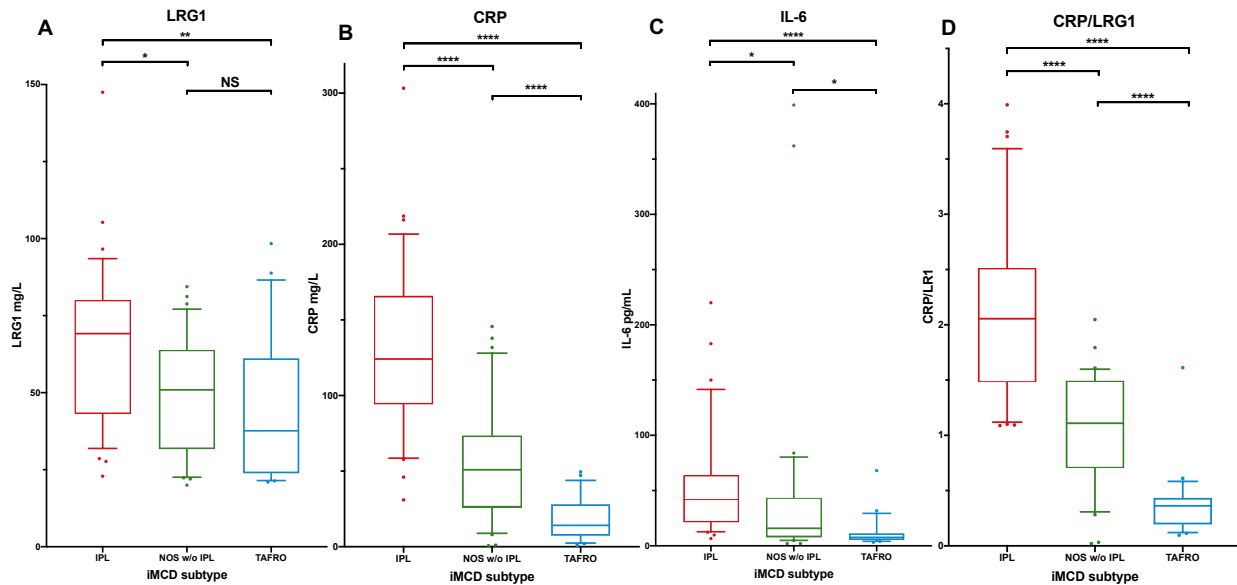

Supplement: Supplementary file 5 — Supplementary Material 5. [file 40364_2025_767_MOESM5_ESM.pdf]
